# Effects of nursing intervention based on the theory of planned behavior on visual function, blood sugar levels, and quality of life in perioperative patients with diabetic retinopathy

**DOI:** 10.3389/fmed.2025.1636854

**Published:** 2025-11-13

**Authors:** Fang Wang, Mengying Shang, Lin Yang, Jing Jiang, Hailian Huang

**Affiliations:** Department of Ophthalmology, The Affiliated Huaian No.1 People’s Hospital of Nanjing Medical University, Huai’an, Jiangsu, China

**Keywords:** diabetic retinopathy, theory of planned behavior, nursing, visual function, blood glucose level

## Abstract

**Objective:**

To explore the impact of nursing intervention based on the Theory of Planned Behavior (TPB) on perioperative patients with diabetic retinopathy (DR).

**Methods:**

One hundred and twenty patients with DR admitted to our hospital from January 2021 to January 2024 were divided into intervention group and control group. The control group received conventional nursing intervention. The intervention group received nursing intervention based on TPB. The visual function, blood sugar levels, incidence of adverse events, self-management ability and quality of life of the two groups were compared using *t*-test or χ^2^ test.

**Results:**

After 2 months of intervention, the peripheral vision score of the intervention group was 5.81 points lower than that before the intervention (*P* < 0.001); the score of sensory adaptation was 9.20 points lower than that before the intervention (*P* < 0.001); the score of daily activity restriction was 9.06 points lower than that before the intervention (*P* < 0.001). After 2 months of intervention, the peripheral vision score in the control group was 1.51 points lower than that before the intervention (*P* < 0.001); the score of sensory adaptation was 5.20 points lower than that before the intervention (*P* < 0.001); the score of daily activity restriction was 5.04 points lower than that before the intervention (*P* < 0.001). Compared with the control group, the intervention group had lower scores on the visual function scale in terms of peripheral vision, sensory adaptation, and daily activity restriction 2 months after the intervention. Specifically, the peripheral vision score of the intervention group was 2.15 points lower than that of the control group (*P* < 0.001); the sensory adaptation score was 3.97 points lower than that of the control group (*P* < 0.001); the score of daily activity restriction was 4.00 points lower than that of the control group (*P* < 0.001). After 2 months of intervention, the fasting blood glucose levels of the intervention group were 3.07 mmol/L lower than those before the intervention (*P* < 0.001); the 2 h postprandial blood glucose levels were 4.22 mmol/L lower than those before the intervention (*P* < 0.001); the glycosylated hemoglobin was 3.78% lower than that before the intervention (*P* < 0.001). After 2 months of intervention, the fasting blood glucose levels of the control group were 1.00 mmol/L lower than those before the intervention (*P* < 0.001); the 2 h postprandial blood glucose levels were 2.19 mmol/L lower than those before the intervention (*P* < 0.001); the glycosylated hemoglobin was lower 1.76% than that before the intervention (*P* < 0.001). Compared with the control group, the intervention group had lower fasting blood glucose levels, 2 h postprandial blood glucose levels, and glycosylated hemoglobin 2 months after the intervention. Specifically, the fasting blood glucose levels of the intervention group were 1.93 mmol/L lower than those of the control group (*P* < 0.001); the 2 h postprandial blood glucose levels were 2.00 mmol/L lower than those of the control group (*P* < 0.001); the glycosylated hemoglobin was 1.99% lower than that of the control group (*P* < 0.001). The incidence of adverse events in the intervention group was 5.01%, and that in the control group was 16.67%. Compared to the control group, the intervention group had a lower incidence of adverse events, with significant difference (*P* = 0.039). After 2 months of intervention, the self-concept score of the intervention group was 4.70 points higher than that before the intervention (*P* < 0.001); the score of self-care responsibility was 5.40 points higher than that before the intervention (*P* < 0.001); the score of self-care ability was 6.34 points higher than that before the intervention (*P* < 0.001); the score of health knowledge level was 6.36 points higher than that before the intervention (*P* < 0.001); the total score was 15.00 points higher than that before the intervention (*P* < 0.001). After 2 months of intervention, the self-concept score of the control group was 2.50 points higher than that before the intervention (*P* < 0.001); the score of self-care responsibility was 2.16 points higher than that before the intervention (*P* < 0.001); the score of self-care ability was 3.98 points higher than that before the intervention (*P* < 0.001); the score of health knowledge level was 3.24 points higher than that before the intervention (*P* < 0.001); the total score was 8.00 points higher than that before the intervention (*P* < 0.001). Compared with the control group, the intervention group had higher scores on the ESCA scores of the two groups in the aspects of self-concept, self-care responsibility, self-care ability, health knowledge level and total score 2 months after the intervention. Specifically, the self-concept score of the intervention group was 2.00 points higher than that of the control group (*P* < 0.001); the self-care responsibility score of the intervention group was 3.22 points higher than that of the control group (*P* < 0.001); the self-care ability score was 2.30 points higher than that of the control group (*P* < 0.001); the health knowledge level score was 3.10 points higher than that of the control group (*P* < 0.001); the total score was 6.00 points higher than that of the control group (*P* < 0.001). After 2 months of intervention, the reading and fine motor score of the intervention group was 10.11 points higher than that before the intervention (*P* < 0.001); the score of adjustment ability was 7.16 points higher than that before the intervention (*P* < 0.001); the score of daily living was 6.57 points higher than that before the intervention (P < 0.001); the score of far vision, movement and light sense was 21.80 points higher than that before the intervention (*P* < 0.001). After 2 months of intervention, the reading and fine motor score of the control group was 4.71 points higher than that before the intervention (*P* < 0.001); the score of adjustment ability was 4.38 points higher than that before the intervention (*P* < 0.001); the score of daily living was 1.81 points higher than that before the intervention (*P* < 0.001); the score of far vision, movement and light sense was 13.74 points higher than that before the intervention (*P* < 0.001). Compared with the control group, the intervention group had higher scores on the higher LVQOL scores in the aspects of reading and fine motor, adjustment ability, daily living, and far vision, movement and light sense 2 months after the intervention. Specifically, the reading and fine motor score of the intervention group was 5.33 points higher than that of the control group (*P* < 0.001); the adjustment ability score was 2.75 points higher than that of the control group (*P* < 0.001); the daily living score was 4.73 points higher than that of the control group (*P* < 0.001); the far vision, movement and light sense score was 8.10 points higher than that of the control group (*P* < 0.001).

**Conclusion:**

Nursing intervention based on TPB can effectively improve the blood glucose control effect of DR patients, improve their visual function, and enhance their self-management ability.

## Introduction

Diabetes is an endocrine and metabolic disorder characterized by persistently elevated blood sugar levels. According to the American Diabetic Association guidelines, the reasonable threshold for “elevated blood sugar levels” is clearly defined as follows: fasting blood glucose remains above 7.0 mmol/L (126 mg/dL); 2-h blood glucose after oral glucose tolerance test remains above 11.1 mmol/L (200 mg/dL); random blood glucose remains above 11.1 mmol/L (200 mg/dL) and accompanied by typical diabetes symptoms (1). With the rapid economic development, the incidence of diabetes worldwide has been continuously increasing, seriously endangering people’s quality of life, and becoming a major public health issue globally (2). It is estimated that by 2045, the number of diabetic patients in China will increase to 700 million (3). The incidence of diabetes-related complications is constantly increasing as the number of diabetes patients rises (4). According to data from the World Health Organization, by 2025, about one-third of all diabetes patients worldwide will develop diabetic retinopathy (DR) (5). DR isan eye condition caused by retinal microvascular damage resulting from chronic progressive diabetes (6). Blurred vision and decreased vision are the main clinical symptoms of DR (7). DR has the characteristics of a high prevalence and a high rate of blindness, which poses a potential threat to public health (8). According to reports, by 2019, the number of cases of blindness caused by DR in China had reached 230,000, and this trend is gradually intensifying (9). The visual impairment caused by DR is often irreversible and frequently leads to eye diseases such as retinal detachment and glaucoma, and even results in severe consequences such as blindness (10). In addition to visual impairment, DR also significantly increases the probability of death from cardiovascular disease deaths and aggravates the economic burden on families and society (11).

At present, most patients with DR undergo surgical treatment to control their condition. Such surgeries can delay the progression of the disease, including intravitreal injections, retinal photocoagulation, and vitrectomy (12). However, before and after the surgery, patients often experience psychological and physiological reactions, which can affect the surgical outcome (13). Therefore, it is necessary to provide adequate perioperative care for patients, with the aim of alleviating their pain, avoiding complications, improving the success rate of the surgical treatment, effectively consolidating the treatment effect, and promoting their recovery (14).

The Theory of Planned Behavior (TPB) was first proposed by American psychologist Ajzen in 1991 (15). This theory starts from the characteristics of individuals themselves, focusing on their background features, social factors, actual conditions and other aspects, and deeply explores the relevant influencing factors and paths during the process of behavior change. Then, targeted intervention measures are taken to enhance belief, reduce resistance, guide individuals to adjust their behaviors, and promote behavioral changes (16).

The nursing intervention measures based on the TPB have rich and specific manifestations in practical applications. Take the nursing care for patients with type 2 diabetes as an example (17). When applying the TPB, the first step is to assess the patients’ attitudes toward disease management and health behaviors (such as proper diet, regular exercise, and taking medication on time). If it is found that the patients have a negative attitude toward regular exercise, believing that it is tiring and has no obvious effect, the nursing staff will use health lectures and sharing of successful cases to show the patients the significant effects of regular exercise on controlling blood sugar and preventing complications, thereby changing the patients’ attitudes toward exercise. Secondly, they will examine the patients’ subjective norms, that is, the degree to which the patients perceive the expectations and support from important others such as family members, friends, and doctors for their health behaviors. If the patients feel that their family members do not support them in controlling their diet, the nursing staff will communicate with the family members to let them understand the importance of a proper diet for diabetes treatment and encourage the family members to participate in the patients’ diet management, creating a good family support environment for the patients and enhancing their subjective norms of following healthy diet behaviors. Finally, they will pay attention to the patients’ perceived behavioral control, that is, the patients’ perception of the difficulty in executing healthy behaviors. For patients who find it troublesome to take medication on time, the nursing staff will provide medication box packaging services, dividing the daily medications into time and dosage groups and setting medication reminders to reduce the difficulty for the patients in executing the medication on time and improve their perceived behavioral control. Through these intervention measures based on the TPB, multiple dimensions are influenced to affect the patients’ behavioral decisions, motivating the patients to actively adopt healthy behaviors conducive to disease control. Traditional nursing intervention measures usually focus more on monitoring the physiological indicators of the disease and basic nursing operations. For example, in the care of patients with type 2 diabetes, traditional nursing mainly involves regularly measuring the patients’ physiological indicators such as blood sugar and blood pressure, guiding patients on the correct use of hypoglycemic drugs, informing patients of some basic dietary precautions, but lacks in-depth exploration and targeted intervention of the psychological process of patients’ behavioral changes. Traditional nursing rarely considers the patients’ true attitudes toward healthy behaviors, the influence of their social environment on their behaviors, and the difficulty they perceive in executing behaviors. In contrast, nursing intervention measures based on the TPB are more comprehensive, in-depth and personalized. It not only focuses on the physiological aspect of the disease, but also pays more attention to the psychological, social and behavioral factors of the patients, and promotes positive behavioral changes through systematically influencing these factors.

In recent years, nursing interventions based on TPB have been widely used in the nursing of a variety of diseases, such as osteoporosis (18), type 2 diabetes (19) and prostate cancer (20). However, the application of nursing interventions based on TPB in perioperative DR patients has rarely been reported. Therefore, the present study aimed to explore the impact of nursing intervention measures based on TPB on perioperative patients with DR.

## Materials and methods

### Study design

This was a prospective intervention study. One hundred and twenty patients with DR admitted to our hospital from January 2021 to January 2024 were selected as the study participants. According to the random number table method, the patients were divided into the intervention group and the control group, with 60 cases in each group. In this study, patients with DR were classified into stages I, II, and III based on the stage of diabetes. The diabetes staging criteria were referred to the guidelines jointly formulated by the World Health Organization (WHO) and the American Diabetes Association (ADA). The clinical registration number was ChiCTR2200060711.

Inclusion criteria: (1) Patients met the diagnostic criteria for DR. According to the “Clinical Guidelines for Diabetic Retinopathy” released by the American Academy of Ophthalmology (AAO) in 2023, as well as the “Clinical Diagnosis and Treatment Guidelines for Diabetic Retinopathy in China (2022)” formulated by the Ocular Fundus Disease Group of the Ophthalmology Branch of the Chinese Medical Association, DR is one of the most common microvascular complications of diabetes. It occurs due to long-term high blood sugar and the combined effects of various other factors, causing leakage and obstruction of retinal microvessels, which leads to ischemia and hypoxia in the retina and triggers a series of retinal pathological changes. This can seriously affect a patient’s vision and even cause blindness; (2) The patient had no contraindications to surgery. For instance, severe uncontrolled hypertension (systolic blood pressure > 180 mmHg, diastolic blood pressure > 110 mmHg), abnormal coagulation function (such as hemophilia causing significant prolongation of clotting time), or active eye infections (such as acute conjunctivitis and keratitis) are all conditions that make surgery unsuitable; (3) This experiment was approved by the Medical Ethics Committee of our hospital, and patients and their family members signed the informed consent. Exclusion criteria: (1) Patients with consciousness disorder or mental illness, where consciousness disorder is defined as a score of ≤ 8 on the Glasgow Coma Scale (GCS); mental illness is determined according to the diagnostic criteria for mental and behavioral disorders in the “Chinese Classification and Diagnostic Criteria for Mental Disorders (CCMD-3)” or the “International Classification of Diseases (ICD - 10)”; (2) Patients combined with malignant tumor; (3) The heart, liver, kidney, and other vital organs suffer from severe dysfunction.

### Nursing intervention

The control group received conventional nursing intervention, which mainly included close monitoring of various indicators of the patients, especially the blood sugar level, following the doctor’s advice and adjusting the dosage of drugs according to the recovery of the patients’ condition, administering routine dietary guidance to the patients and carrying out simple health education. For some patients with obvious vision loss, the accompanying care was strengthened to prevent the occurrence of falling out of bed and injuries.

The observation group received nursing interventions based on TPB.

(1)    Admission assessment: DR questionnaire was designed according to illness status and educational level, including subjective intention, health behavior, and perceived control.
(2)    Preoperative behavioral intervention. Preoperative behavioral intervention included behavioral attitude intervention and normative subjective behavior. Behavioral attitude intervention: The nurse used methods such as oral explanations, video demonstrations, and on-site guidance to enable the patient to understand the disease, the treatment process, and its effects. The nurse took the initiative to patiently answer the patient’s doubts, and appropriately restricted the patient’s head movement to prevent the hole from affecting the retinal traction. At the same time, the nurse told the patient not to sneeze and cough vigorously, guided the correct way, made adequate preparations for the operation, avoided the occurrence of adverse conditions, and ensured the patient’s behavior and health. Normative subjective behavior: The patients were repeatedly explained in detail about the treatment prospects and the significance of the disease. The nurse recorded the patient’s condition and operation method, and actively asked the attending physician about the patient’s current condition and precautions linked to the operation to ensure that each nursing service was targeted. The nurse invited the patients to participate in the health knowledge lecture, mainly explaining the importance of preoperative psychological balance, how to adjust their emotions, and guiding the patients through psychological suggestion, attention transfer and stress relief. At the same time, nurses used successful cases to enhance patients’ cooperation and understanding, and constantly standardized patients’ surgical cognition.
(3)    Intraoperative coordination. The nurse prepared surgical items and instruments, calmed the patient’s emotions, and told him/her to strictly follow the doctor’s advice to regulate his own behavior during the operation, so as to avoid emotional overreaction causing stress. At the same time, the nurse cooperated with the doctor to carry out relevant operations to improve the efficiency of the operation. During this period, the nurse paid close attention to the vital signs of the patients, and timely reacted to any adverse conditions and gave corresponding operations to ensure the smooth operation.
(4)    Postoperative behavior control. Postoperative behavior control included dietary guidance, eye care, exercise instruction, and postoperative follow-up. Dietary guidance: Based on age, height, and body mass, the nurse calculated energy needs and created a personalized eating plan, recommending small, frequent meals and a balanced diet rich in high-quality protein, vegetables, and fruits, while supplementing vitamins and trace elements. Foods high in sugar, fat, and spicy or irritating ingredients were prohibited. Eye care: The nurse instructed patients on self-monitoring, medication compliance for blood pressure control, and avoiding prolonged screen use and staying up late. Patients were advised to return promptly in case of eye discomfort to prevent complications. Exercise instruction: Patients were advised to engage in moderate aerobic exercises like jogging, climbing, cycling, and taijiquan for about 30 min per session, avoiding strenuous activities and fasting-state workouts to prevent elevated intraocular pressure. Postoperative follow-up: The nurses conducted 2–3 phone follow-ups each month to assess the patient’s recovery progress and address any related issues. Additionally, the nurses made home visits to provide guidance on eye care, diet, and exercise, to help the patient overcome the difficulties they encounter.

All patients in two groups were cared for 2 months.

### Sample selection

Power analysis was carried out using G*Power 3.1.9.7 software to determine the sample size required to detect statistical differences. Based on previous studies on the impact of diabetes care interventions on blood glucose levels (21), it was found that similar intervention measures typically have an effect size ranging from 0.5 to 0.7 in improving the blood glucose levels of diabetic patients. Therefore, the effect size of the indicators related to blood glucose control was set at 0.6. With an alpha level of 0.05 and 90% power, the research revealed that a sample size of 60 patients per group was required.

### Parameters to measure with respective tools

#### Visual function

Chinese version visual function index-14 (VF-14-CN) was used to evaluate the improvement of visual function of patients (22), which included three dimensions: peripheral visual field (0–10 points), sensory adaptation (0–30 points), and daily activity restriction (0–30 points). Under the guidance of the same ophthalmologist, patients completed the questionnaire, affirming whether their daily activities were impacted by visual function impairment, accounting for correction with glasses. Responses range from “no difficulty” to “unable to do the activity” across five response categories and scored on a scale from 0 to 4, respectively. The lower the score, the better the improvement of visual function was.

#### Self-management ability

Using the self-care agency scale (ESCA) (23), the self-management ability between the two groups was assessed. The ESCA was translated and adapted into the Chinese-version ESCA. The Cronbach’s α coefficient of the ESCA scale in this study was 0.84. The full score was 172, and the score was proportional to the self-care ability.

#### Quality of life

Utilizing the Low Vision Quality of Life (LVQOL) (24), the quality of life was assessed. The LVQOL was translated and adapted into the Chinese-version LVQOL, including 25 items related to vision decline, which were divided into 4 dimensions, including reading and fine motor (0–25 points), adjustment ability (0–20 points), daily living (0–20 points), far vision, movement and light sense (0–60 points), with a total score of 125 points. The Cronbach’s alpha coefficient and the split-half coefficient for the four scales and total Chinese-version LVQOL scales were 0.75–0.97. The higher the score, the better the quality of life was.

### Data collection

The fasting blood glucose and 2 h postprandial blood glucose levels were detected using a blood glucose meter. A total of 3 mL of fasting venous blood was collected from the patients, and the percentage value of glycosylated hemoglobin was detected using a glycosylated hemoglobin analyzer.

The incidence of adverse events of two groups was compared, including infection, intraocular hypertension, and vitreous hemorrhage.

### Data analysis

Data in this study were analyzed and processed by SPSS 20.0 statistical software. Measurement data expressed as (x ± s), and a paired *t*-test was used for the pre-post comparison of each group, and an independent *t*-test was used for the comparison between the two groups. Count data expressed as rate (%) were compared using χ^2^ test. P < 0.05 was considered statistically significant.

## Results

### Baseline characteristics of patients between the two groups

The intervention group contained 34 males and 26 females, ranging in age from 41 to 70 years, with an average age of (54.51 ± 8.17) years. The control group contained 33 males and 27 females, ranging in age from 42 to 71 years, with an average age of (55.53 ± 8.46) years. There were no differences in the basic characteristics of patients such as gender, age, course of disease and diabetes staging between the two groups (*P* > 0.05) (Table 1).

**TABLE 1:** Baseline characteristics of patients between the two groups.

Items	Intervention group (*n* = 60)	Control group (*n* = 60)	χ^2^/t
Gender			0.033
Male	34 (56.67)	33 (55.00)	
Female	26 (43.33)	27 (45.00)	
Age (years)	54.51 ± 8.17	55.53 ± 8.46	0.671
Course of disease (years)	1.46 ± 0.16	1.48 ± 0.19	0.623
Diabetes staging			0.208
Stage I	26 (43.33)	25 (41.67)	
Stage II	23 (38.33)	22 (36.67)	
Stage III	11 (18.34)	13 (21.66)	

### Visual function between the two groups

**FIGURE 1:**
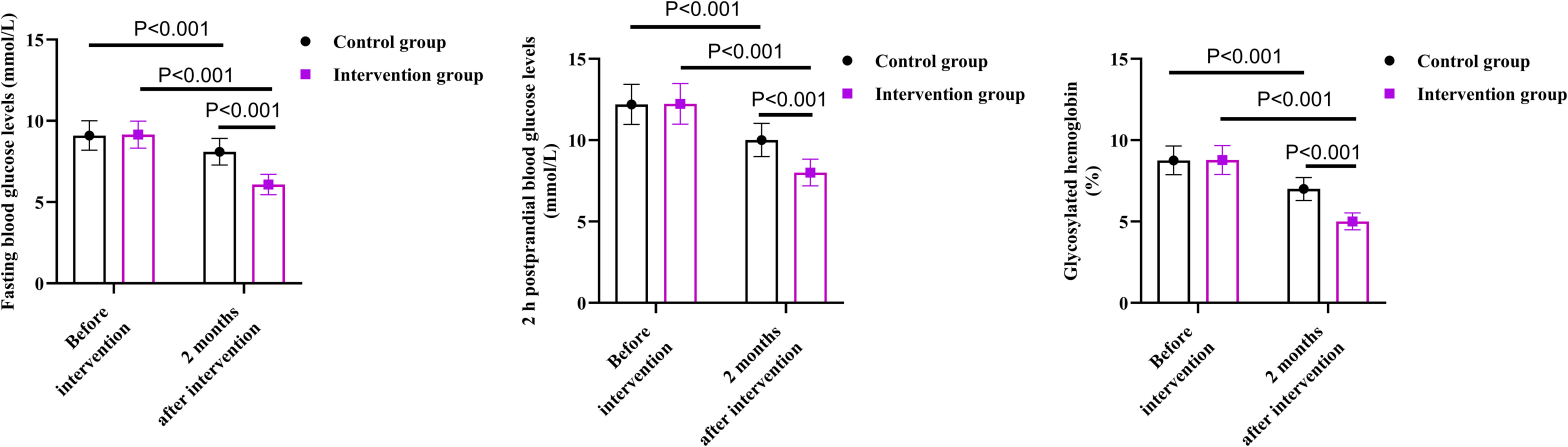
Visual function between the two groups.

As shown in Figure 1, before the intervention, there were no differences in visual function scale scores of the two groups in terms of peripheral visual field, sensory adaptation, and daily activity restriction (*P* = 0.863, *P* = 0.929, *P* = 0.950).

After 2 months of intervention, the peripheral vision score of the intervention group was 5.81 points lower than that before the intervention (*P* < 0.001); the score of sensory adaptation was 9.20 points lower than that before the intervention (*P* < 0.001); the score of daily activity restriction was 9.06 points lower than that before the intervention (*P* < 0.001).

After 2 months of intervention, the peripheral vision score in the control group was 1.51 points lower than that before the intervention (*P* < 0.001); the score of sensory adaptation was 5.20 points lower than that before the intervention (*P* < 0.001); the score of daily activity restriction was 5.04 points lower than that before the intervention (*P* < 0.001).

Compared with the control group, the intervention group had lower scores on the visual function scale in terms of peripheral vision, sensory adaptation, and daily activity restriction 2 months after the intervention. Specifically, the peripheral vision score of the intervention group was 2.15 points lower than that of the control group (*P* < 0.001); the sensory adaptation score was 3.97 points lower than that of the control group (*P* < 0.001); the score of daily activity restriction was 4.00 points lower than that of the control group (*P* < 0.001).

### Blood sugar levels between the two groups

**FIGURE 2:**
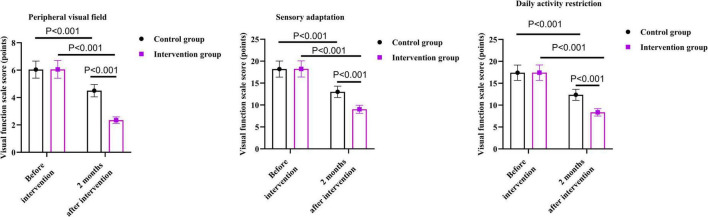
Blood sugar levels between the two groups.

As shown in Figure 2, before the intervention, there were no differences in the fasting blood glucose levels, 2 h postprandial blood glucose levels, and glycosylated hemoglobin between the two groups (*P* = 0.753, *P* = 0.894, *P* = 0.801).

After 2 months of intervention, the fasting blood glucose levels of the intervention group were 3.07 mmol/L lower than those before the intervention (*P* < 0.001); the 2 h postprandial blood glucose levels were 4.22 mmol/L lower than those before the intervention (*P* < 0.001); the glycosylated hemoglobin was 3.78% lower than that before the intervention (*P* < 0.001).

After 2 months of intervention, the fasting blood glucose levels of the control group were 1.00 mmol/L lower than those before the intervention (*P* < 0.001); the 2 h postprandial blood glucose levels were 2.19 mmol/L lower than those before the intervention (*P* < 0.001); the glycosylated hemoglobin was lower 1.76% than that before the intervention (*P* < 0.001).

Compared with the control group, the intervention group had lower fasting blood glucose levels, 2 h postprandial blood glucose levels, and glycosylated hemoglobin 2 months after the intervention. Specifically, the fasting blood glucose levels of the intervention group were 1.93 mmol/L lower than those of the control group (*P* < 0.001); the 2 h postprandial blood glucose levels were 2.00 mmol/L lower than those of the control group (*P* < 0.001); the glycosylated hemoglobin was 1.99% lower than that of the control group (*P* < 0.001).

### Incidence of adverse events between the two groups

As shown in Table 2, the incidence of adverse events in the intervention group was 5.01%, and that in the control group was 16.67%. Compared to the control group, the intervention group had a lower incidence of adverse events, with significant difference (*P* = 0.039).

**TABLE 2:** Incidence of adverse events between the two groups.

Groups	Case	Infection	Intraocular hypertension	Vitreous hemorrhage	Total incidence rate
Intervention group	60	1 (1.67)	1 (1.67)	1 (1.67)	3 (5.01)
Control group	60	3 (5.00)	4 (6.67)	3 (5.00)	10 (16.67)
χ^2^					4.227
*P*					0.039

### Self-management ability between the two groups

**FIGURE 3:**
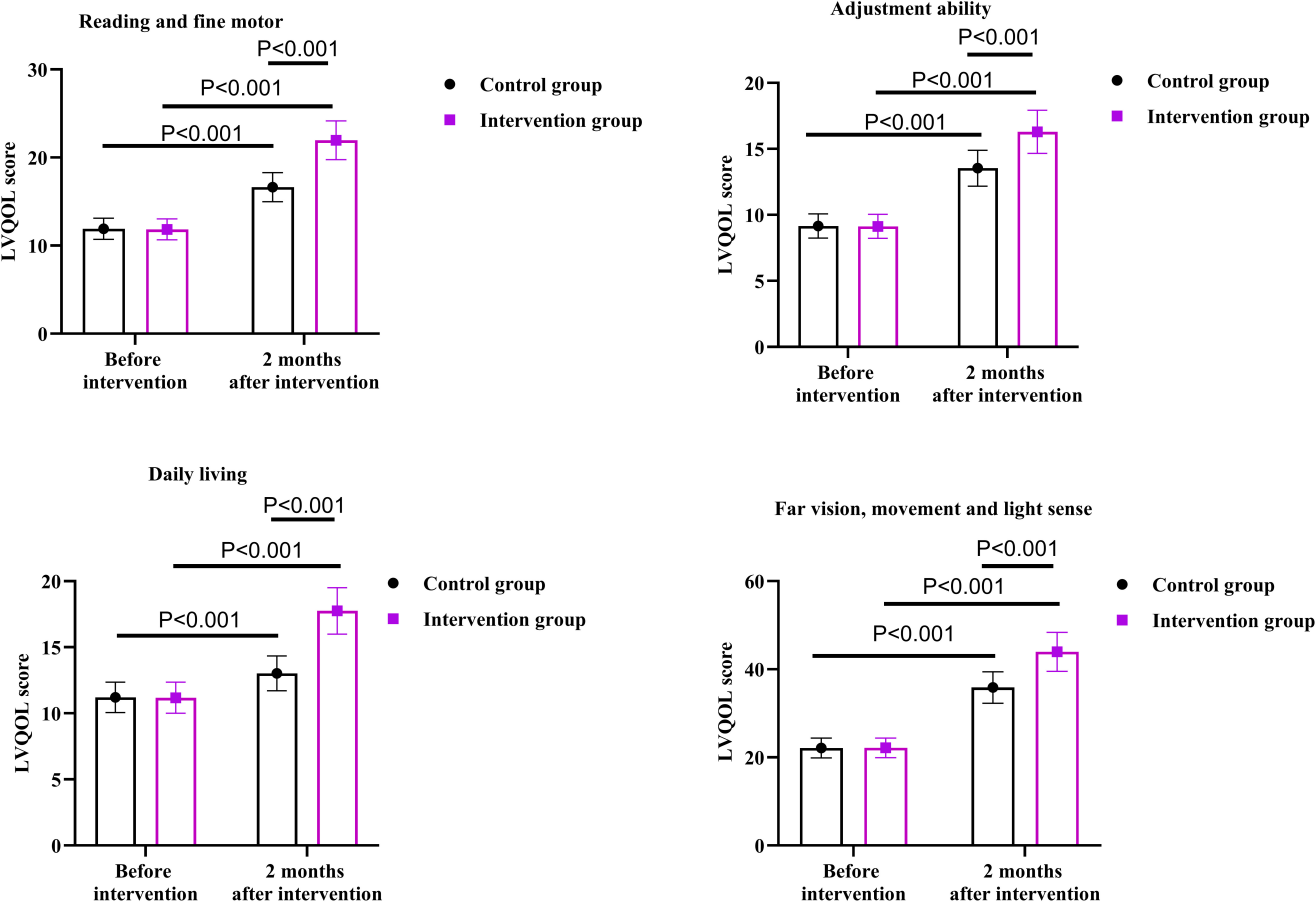
Self-management ability between the two groups.

As shown in Figure 3, before the intervention, there were no differences in ESCA scores of the two groups in the aspects of self-concept, self-care responsibility, self-care ability, health knowledge level and total score (*P* = 0.526, *P* = 0.929, *P* = 0.900, *P* = 0.981, *P* = 0.631).

After 2 months of intervention, the self-concept score of the intervention group was 4.70 points higher than that before the intervention (*P* < 0.001); the score of self-care responsibility was 5.40 points higher than that before the intervention (*P* < 0.001); the score of self-care ability was 6.34 points higher than that before the intervention (*P* < 0.001); the score of health knowledge level was 6.36 points higher than that before the intervention (*P* < 0.001); the total score was 15.00 points higher than that before the intervention (*P* < 0.001).

After 2 months of intervention, the self-concept score of the control group increased by 2.50 points than that before the intervention (*P* < 0.001); the score of self-care responsibility was 2.16 points higher than that before the intervention (*P* < 0.001); the score of self-care ability was 3.98 points higher than that before the intervention (*P* < 0.001); the score of health knowledge level was 3.24 points higher than that before the intervention (*P* < 0.001); the total score was 8.00 points higher than that before the intervention (*P* < 0.001).

Compared with the control group, the intervention group had higher scores on the ESCA scores of the two groups in the aspects of self-concept, self-care responsibility, self-care ability, health knowledge level and total score 2 months after the intervention. Specifically, the self-concept score of the intervention group was 2.00 points higher than that of the control group (*P* < 0.001); the self-care responsibility score of the intervention group was 3.22 points higher than that of the control group (*P* < 0.001); the self-care ability score was 2.30 points higher than that of the control group (*P* < 0.001); the health knowledge level score was 3.10 points higher than that of the control group (*P* < 0.001); the total score was 6.00 points higher than that of the control group (*P* < 0.001).

### Quality of life between the two groups

**FIGURE 4:**
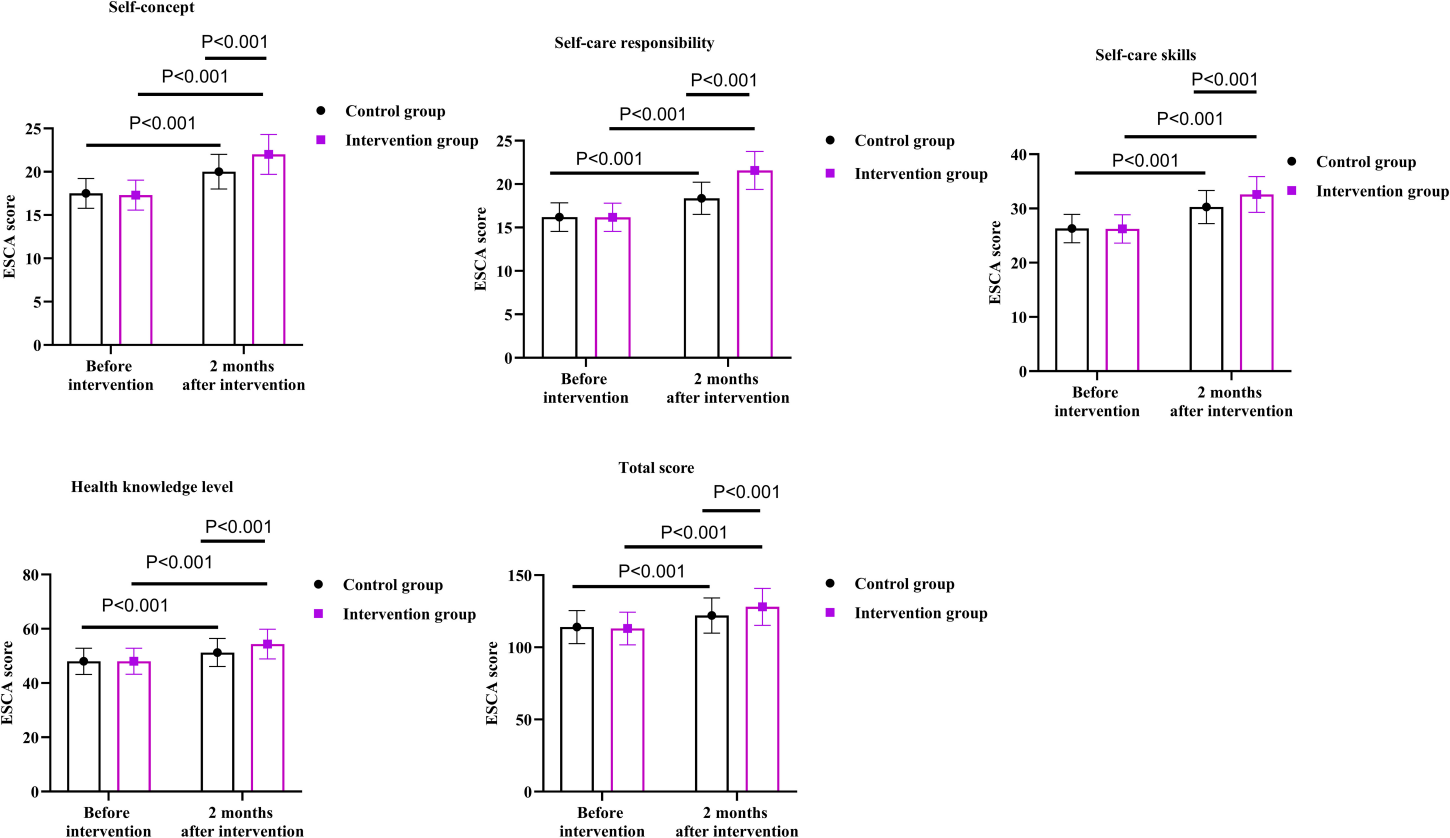
Quality of life between the two groups.

As shown in Figure 4, before the intervention, there were no differences in LVQOL scores between the two groups in the aspects of reading and fine motor, adjustment ability, daily living, and far vision, movement and light sense (*P* = 0.747, *P* = 0.857, *P* = 0.888, *P* = 0.922).

After 2 months of intervention, the reading and fine motor score of the intervention group was 10.11 points higher than that before the intervention (*P* < 0.001); the score of adjustment ability was 7.16 points higher than that before the intervention (*P* < 0.001); the score of daily living was 6.57 points higher than that before the intervention (*P* < 0.001); the score of far vision, movement and light sense was 21.80 points higher than that before the intervention (*P* < 0.001).

After 2 months of intervention, the reading and fine motor score of the control group was 4.71 points higher than that before the intervention (*P* < 0.001); the score of adjustment ability was 4.38 points higher than that before the intervention (*P* < 0.001); the score of daily living was 1.81 points higher than that before the intervention (*P* < 0.001); the score of far vision, movement and light sense was 13.74 points higher than that before the intervention (*P* < 0.001).

Compared with the control group, the intervention group had higher scores on the higher LVQOL scores in the aspects of reading and fine motor, adjustment ability, daily living, and far vision, movement and light sense 2 months after the intervention. Specifically, the reading and fine motor score of the intervention group was 5.33 points higher than that of the control group (*P* < 0.001); the adjustment ability score was 2.75 points higher than that of the control group (*P* < 0.001); the daily living score was 4.73 points higher than that of the control group (*P* < 0.001); the far vision, movement and light sense score was 8.10 points higher than that of the control group (*P* < 0.001).

## Discussion

This study aimed to explore the impact of nursing intervention measures based on TPB on perioperative patients with DR. The results indicated that nursing intervention based on TPB can effectively improve the blood glucose control effect of DR patients, reduce the incidence of adverse events, improve their visual function, and enhance their self-management ability.

DR is an ophthalmic disease characterized by changes in vascular microcirculation and tissues due to abnormal insulin metabolism in patients, thereby damaging the patient’s eye functions (25). Clinical surgery is mainly used to control the continuous decline of visual acuity (26). Although routine perioperative nursing interventions can improve the postoperative visual function of DR patients to a certain extent, due to the existence of individual differences among patients, this nursing model is difficult to exert targeted effects, resulting in significant differences in efficacy and inability to achieve good nursing results (27).

Nursing intervention based on TPB is a nursing model guided by TPB. The TPB is a theoretical model consisting of five elements: behavioral attitude, subjective norms, perceived behavioral control, behavioral willingness and behavior (18). It believes that behavior is controlled by behavioral attitude, subjective norms and perceived behavior, and has been widely used in research in many fields of research (28). With the development of medical technology, TPB has been gradually applied in the field of nursing in recent years (29). Compared with routine nursing interventions, the nursing intervention based on TPB has the following significant advantages and differences: (1) Personalization and targeted approach: Standardized routine care interventions typically follow standardized procedures and measures, applying the same care plan to all patients, which is difficult to fully consider the individual differences of patients, such as cognitive level, psychological state, and lifestyle. However, the nursing intervention based on TPB is tailored to the cognitive level, physical and psychological conditions of patients. (2) Guidance for Behavior Change: Routine care interventions may focus more on the physiological treatment and nursing operations of the disease, and provide relatively less guidance for patients’ behavior change. The nursing intervention based on TPB emphasizes the key role of behavior change in preventing and controlling diabetes. By starting from aspects such as patients’ behavioral attitudes, subjective norms, and perceived behavioral control, it stimulates patients’ intrinsic motivation and prompts them to actively change unhealthy behavioral habits. After surgery, through intervention measures such as emotion control, eye care, exercise and diet, patients’ unhealthy behavioral habits are effectively corrected, promoting the formation of healthy behaviors, thereby improving vision and controlling blood sugar levels. (3) Comprehensiveness and systematicness: Routine care interventions may focus on a certain aspect or link of care, lacking comprehensiveness and systematicness. The nursing intervention based on TPB covers all aspects of the patient’s perioperative period, including preoperative education, intraoperative cooperation, postoperative care and follow-up guidance. During the surgery, in close cooperation with the doctor, make adequate preparations to improve the safety of the surgery and reduce the stress response of patients. After the surgery, provide eye care, exercise guidance and diet advice for patients, and through follow-up guidance, improve patients’ self-care ability to achieve the goal of preventing adverse events. This comprehensive and systematic nursing approach can better promote the recovery of patients.

In China, the standard nursing intervention measures for patients with DR mainly include general care, drug treatment care, preoperative and postoperative care and health education. However, as mentioned earlier, these standard nursing intervention measures have certain limitations in addressing individual differences of patients and guiding behavioral changes, while the nursing intervention based on TPB can make up for these deficiencies and achieve better nursing outcomes.

In our study, the results showed that compared to the control group, the intervention group had lower visual function scale scores in the aspects of peripheral visual field, sensory adaptation, and daily activity restriction 2 months after the intervention. Additionally, the fasting blood glucose level, 2 h postprandial blood glucose level, and glycosylated hemoglobin level of the intervention group were also lower after 2 months of the intervention. Therefore, nursing intervention based on TPB could effectively improve the blood glucose levels of patients with DR and promote the recovery of visual function, which was consistent with the results of previous studies (30). This is because in the treatment process of patients with DR, behavioral changes are a crucial step in preventing and controlling diabetes. Based on the theory of TPB, a targeted nursing intervention was a set of measures tailored to the patient’s cognitive level, physical and mental conditions. Firstly, it was carried out from the aspect of the patient’s behavioral attitude. Before the surgery, targeted education was provided to enhance the patient’s understanding of the disease, correct their incorrect cognition, and establish a positive behavioral attitude, enabling them to actively cooperate with subsequent treatment and care. Therefore, this is of positive significance for controlling blood sugar and restoring visual function. After the surgery, intervention measures such as emotional control, eye care, exercise, and diet could effectively correct the patient’s unhealthy health behaviors, which not only promoted the development of their healthy behaviors but also improved visual function and controls blood sugar levels.

The results of our study also demonstrated that the incidence of adverse events in the intervention group was lower than that in the control group, suggesting that the nursing intervention based on TPB could improve the safety of the surgery by closely cooperating with the doctors during the operation and making adequate preparations before the surgery, thereby reducing the stress response of the patients. At the same time, postoperative eye care, exercise and diet guidance were provided according to specific circumstances, enabling patients to maintain a healthier lifestyle, thus effectively avoiding adverse risks. By providing postoperative follow-up guidance on how to perform self-care, nurses could enhance patients’ self-care abilities and achieve the goal of preventing adverse event.

Moreover, our study indicated that relative to the control group, the intervention group had lower visual function scale scores in the aspects of peripheral visual field, sensory adaptation, and daily activity restriction and higher LVQOL scores in the aspects of reading and fine motor, adjustment ability, daily living, far vision, movement and light sense All these results indicated that nursing intervention based on TPB could effectively promote the self-management ability and quality of life of DR patients. Consistently, Göger et al. suggested that the training program provided according to TPB could promote the self-efficacy of type 2 diabetes mellitus patients (31). Kamalian et al. indicated that educational intervention lifestyle based on TPB significantly increased the mean scores of quality of life of middle-aged women (32). During the nursing intervention based on TPB, patients were invited to attend health knowledge lectures, which not only enhanced patients’ cognition of the disease and other related knowledge, but also stimulated their intrinsic motivation, strengthened their awareness of perceived behavioral control and behavioral change, and ultimately promoted positive behavioral changes and effectively improved their self-management ability and quality of life. At the same time, under the guidance of nurses, patients could more clearly recognize their incorrect behaviors and actively form good living habits, which helped to improve their self-management ability and quality of life.

Regarding suggestions on how to incorporate these intervention measures into the existing patient care process, we believe that efforts can be made from the following aspects. During the planning stage of the care process, a multidisciplinary team should be organized, including ophthalmologists, nurses, nutritionists, psychological counselors, etc., to jointly participate in the design of the TPB-based nursing intervention plan, ensuring that the intervention measures can comprehensively cover all the needs of patients during the perioperative period and integrate with the existing care process.

During the implementation of the nursing process, the intervention measures based on TPB will be detailed into daily nursing work. When nurses carry out routine nursing operations, they will simultaneously implement behavioral interventions, such as explaining to patients the importance of the medication for blood sugar control and eye recovery when administering the medication, and strengthening the patients’ behavioral intentions. At the same time, by using information technology means, such as developing a specialized nursing APP, to provide patients with real-time health knowledge push, behavior reminders and self-monitoring functions, it is convenient for patients to continue receiving intervention outside the hospital, improving the continuity and effectiveness of the nursing.

In terms of nursing quality monitoring, a specialized evaluation index system based on TPB nursing intervention was established. Regular evaluations and analyses of the intervention effects were conducted. By collecting patients’ feedback, the intervention measures were adjusted in a timely manner to continuously optimize their compatibility with the existing nursing procedures. Additionally, training was strengthened for nursing staff to familiarize them with the TPB-based nursing concepts and methods, and to enhance their ability to apply these intervention measures in actual work. Thus, the TPB-based nursing intervention could be smoothly incorporated into the existing patient care process, providing DR patients with higher-quality and more targeted nursing services.

Our study has some limitations. In terms of sample selection, the number of patients included in this study was relatively limited, and the sample sources mainly concentrated on DR patients from a specific region or medical institution. This may result in insufficient representativeness of the sample and limit the extrapolation of the research results. DR patients from different regions, different races, and different social economic backgrounds may have differences in behavioral attitudes and subjective norms. The effect of the nursing intervention based on TPB in these patients may be different from the results of this study. In terms of the duration of the study, the intervention and observation period of this research was 2 months, which was relatively short. DR is a chronic disease, and its treatment and rehabilitation process usually requires a longer time. A shorter study duration may not be sufficient to fully observe the sustained effects of the nursing intervention based on TPB on patients’ long-term blood sugar control, visual function recovery, and self-management ability.

For future research, it is first recommended to increase the sample size and adopt a multi-center, randomized controlled study design, including patients with DR from different regions, different races, and different socioeconomic backgrounds, in order to enhance the representativeness of the sample and the generalizability of the research results. Through multi-center research, a better understanding of the differences in the effects of TPB-based nursing interventions among different patient groups can be achieved, providing a basis for formulating more targeted nursing plans. Secondly, extend the research period and conduct long-term follow-up studies. This approach will enable a more comprehensive and in-depth assessment of the impact of the nursing intervention based on TPB on the perioperative and long-term recovery of DR patients, including the long-term stability of blood sugar control, the continuous improvement of visual function, and the long-term maintenance of self-management ability, etc. Long-term follow-up studies are helpful in revealing the long-term benefits of the nursing intervention and providing more solid evidence support for clinical nursing practice. Furthermore, future research can also further explore the combined application effects of nursing interventions based on TPB with other nursing models or treatment methods. For instance, integrating the nursing intervention based on TPB with new drug treatments, physical therapies, etc., to observe whether a synergistic effect can be achieved, thereby further improving the treatment efficacy and quality of life of patients with DR. At the same time, in-depth research should be conducted on the specific implementation strategies and optimal intervention timing of the nursing intervention based on TPB at different stages (such as before, during, and after surgery and in different time periods after surgery), in order to optimize the nursing plan and enhance the effectiveness and targeting of the nursing intervention.

## Conclusion

Nursing intervention based on TPB can effectively improve the blood glucose control effect of DR patients, improve their visual function, and enhance their self-management ability.

## Data Availability

The datasets presented in this study can be found in online repositories. The names of the repository/repositories and accession number(s) can be found in the article/supplementary material.
